# Supplementary material for: Chaperone-Like Activity of HSPB5: The Effects of Quaternary Structure Dynamics and Crowding
Source: Int J Mol Sci. 2020 Jul 13;21(14):4940. doi: 10.3390/ijms21144940 (PMC7404038; doi:10.3390/ijms21144940)
Supplement: Supplementary file 1 [file ijms-21-04940-s001.pdf]

International Journal of
*Molecular Sciences*

MDPI

*Supplementary materials*

# Chaperone-like activity of HSPB5: the effects of quaternary structure dynamics and crowding

**Natalia A. Chebotareva *, Svetlana G. Roman, Vera A. Borzova, Tatiana B. Eronina, Valeriya V. Mikhaylova and Boris I. Kurganov**

Bach Institute of Biochemistry, Federal Research Centre "Fundamentals of Biotechnology" of the Russian Academy of Sciences, Leninsky pr. 33, Moscow, 119071, Russia; svetabaj@gmail.com (S.G.R.); squsiebox@gmail.com (V.A.B.), eronina@inbi.ras.ru (T.B.E.); mikhaylova.inbi@inbox.ru (V.V.M.); boris@kurganov.com (B.I.K.)

* Correspondence: n.a.chebotareva@gmail.com

## S1. Asymmetrical Flow Field-Flow Fractionation (AF4) of α-Lactalbumin (αLa)

The AF4 study was performed using Eclipse 3 separation system (Wyatt Technology Corporation, Santa Barbara, CA, USA) with Agilent HPLC pump (Agilent Technologies, Santa Clara, CA, USA). The AF4 channel length was 21.4 cm, channel height 0.35 mm channel spacer. The cellulose membrane (Wyatt Technology Corporation, Santa Barbara, CA, USA) with MWCO 5 kDa was used. The elution system was equipped by UV detector (Agilent Technologies, Santa Clara, CA, USA), MALS detector DAWN HELEOS II (Wyatt Technology Corporation, USA) and RI detector Optilab T-rEX (Wyatt Technology Corporation, Santa Barbara, CA, USA). The preheated at 37 °C αLa samples containing 20 mM DTT were cooled in ice and subsequently analyzed by AF4 at room temperature (22-25 °C). The flow rates were 1) axial flow 1 mL/min, focus flow 3 mL/min for sample focusing stage, 2) axial flow 1 mL/min, cross-flow 6.5 mL/min for sample elution. The elution buffer was 0.1 M Na-phosphate, pH 6.8, containing 3 mM $NaN_3$.

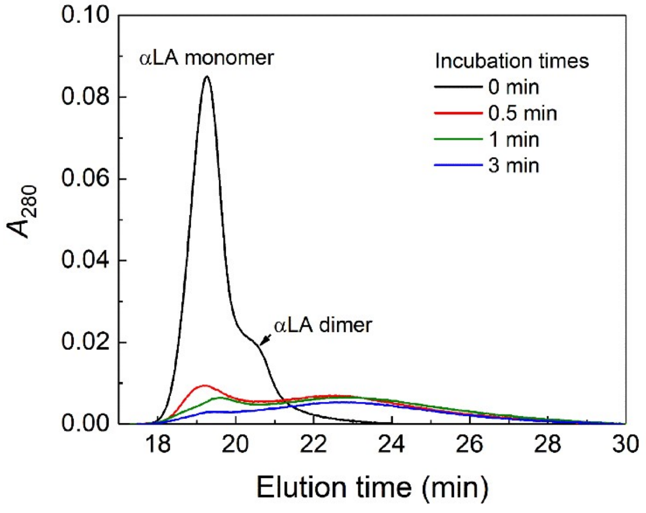


**Figure S1.** The elution profile for αLA preheated at 37 °C in the presence of 20 mM DTT (0.1 M Na-phosphate buffer, pH 6.8) obtained by asymmetric flow field-flow fractionation (AF4). The elution was performed using the Eclipse 3 separation system (Wyatt Technology Corporation, USA) based on an Agilent HPLC pump (Agilent Technologies, USA).

## S2. Particle size characterization in the course of DTT-induced aggregation of αLa in the absence or presence of HSPB5

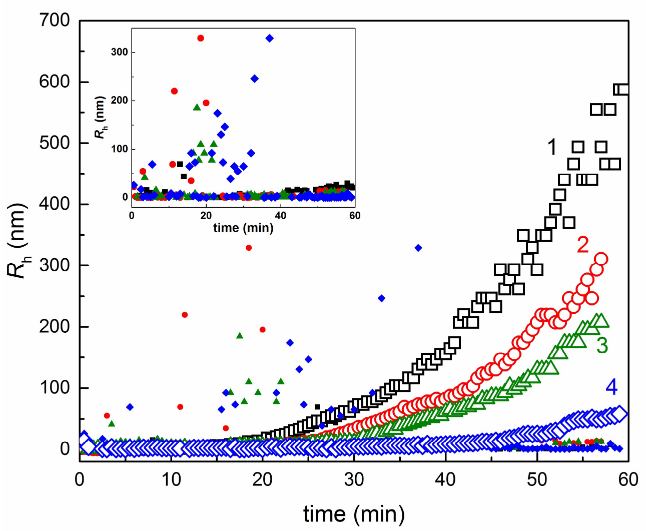


**Figure S2.** The dependence of the hydrodynamic radius ($R_h$) of protein aggregates on time for DTT-induced aggregation of αLa (1 mg/mL) in the absence of HSPB5 (1) and in the presence of 0.0005 mg/mL (2), 0.0075 mg/mL (3) and 0.025 mg/mL (4) of HSPB5. Inset shows the minor population of particles (in colors corresponding to the curves on main panel). The values of $R_h$ were calculated with the following parameters: refractive index, $n$ = 1.33286, dynamic viscosity, η = 0.7273 mPa·s.

The minor population shown on the inset in Figure S2 can be attributed to HSPB5 oligomers in the case of relatively big and polydisperse particles on the initial parts of kinetic curves, or to the smaller αLa aggregates with lower propensity to aggregate or later formation times. The fact that the polydisperse particles disappear later in the course of aggregation may be explained by HSPB5 dissociation and interaction with αLa. Combined with the clearly visible aggregation suppression even in the presence of minimal HSPB5 concentration used, this whole picture may imply the formation of HSPB5–αLa complexes containing very small amount of HSPB5.

## S3. Determination of the Refractive Index, Density and Dynamic Viscosity of the Buffer, Ficoll, PEG and PVP Solutions

The values of the refractive index of 0.03 M Hepes buffer, pH 6.8, containing 0.1 M NaCl, 0.2 mM EDTA, and the refractive index of the same solution containing $Ficoll_{70kDa}$ 75 mg/mL, $PEG_{20kDa}$ 25 mg/mL, $PVP_{10kDa}$ 25 mg/mL, $PVP_{25kDa}$ 25 mg/mL, or their mixtures were determined in ABBEMAT 500 refractometer (“Anton Paar”, Graz, Austria) at 48 °C.

The density of 0.03 M Hepes buffer, pH 6.8, containing 0.1 M NaCl, 0.2 mM EDTA and the densities of the same solution containing $Ficoll_{70kDa}$ 75 mg/mL, $PEG_{20kDa}$ 25 mg/mL, $PVP_{10kDa}$ 25 mg/mL, $PVP_{25kDa}$ in the concentration of 12.5 or 25 mg/mL, or their mixtures were determined in the density meter DMA 4500 (“Anton Paar”, Graz, Austria) at 48 °C.

The dynamic viscosity of 0.03 M Hepes-NaOH buffer, pH 6.8, containing 0.1 M NaCl, 0.2 mM EDTA and dynamic viscosities of the same solution containing $Ficoll_{70kDa}$ 75 mg/mL, $PEG_{20kDa}$ 25 mg/mL, $PVP_{10kDa}$ 25 mg/mL, $PVP_{25kDa}$ in the concentration of 12.5 or 25 mg/mL, or their mixtures were determined in the automated microviscosimeter AMVn (“Anton Paar”, Austria) in system 1.6/1.500 mm at 48 °C.

**Table S1.** The values of the refractive index ($n$), density (ρ) and dynamic viscosity (η) of PEG, Ficoll and PVP solutions at 48 °C (0.03 M Hepes buffer, 0.1 M, NaCl, 0.2 mM EDTA, pH 6.8).

| **Species** | **refractive index ($n$)** | **density (ρ), g/cm³** | **dynamic viscosity (η), mPa·s** |
|---|---|---|---|
| Buffer | 1.33156±0.00002 | 0.99557±0.00007 | 0.5824±0.0007 |
| $PEG_{20kDa}$ 25 mg/mL | 1.33456±0.00003 | 0.99902±0.00015 | 1.231±0.005 |

| | | | |
|---|---|---|---|
| PVP$_{10kDa}$ 25 mg/mL | 1.33557±0.00005 | 1.00015±0.00001 | 0.7027±0.0009 |
| PVP$_{25kDa}$ 12.5 mg/mL | - | 1.00071±0.00005 | 0.7332±0.0025 |
| PVP$_{25kDa}$ 25 mg/mL | 1.33700±0.00005 | 1.00324±0.00004 | 0.9085±0.0008 |
| Ficoll$_{70kDa}$ 75 mg/mL | 1.34139±0.00006 | 1.01919±0.00001 | 1.1827±0.0018 |
| PEG$_{20kDa}$ 25 mg/mL +PVP$_{10kDa}$ 25 mg/mL | 1.33876±0.00006 | 1.00384±0.00007 | 1.393±0.005 |
| PEG$_{20kDa}$ 25 mg/mL +PVP$_{25kDa}$12.5 mg/mL | - | 1.00442±0.00004 | 1.4445±0.0016 |
| PEG$_{20kDa}$ 25 mg/mL +PVP$_{25kDa}$ 25 mg/mL | 1.34006±0.00013 | 1.00668±0.00002 | 1.665±0.003 |
| Ficoll$_{70kDa}$ 75 mg/mL +PEG$_{20kDa}$ 25 mg/mL | 1.34452±0.00006 | 1.02202±0.00008 | 2.158±0.004 |
| Ficoll$_{70kDa}$ 75 mg/mL +PVP$_{10kDa}$ 25 mg/mL | 1.34597±0.00008 | 1.02410±0.00006 | 1.471±0.003 |
| Ficoll$_{70kDa}$ 75 mg/mL +PVP$_{25kDa}$ 25 mg/mL | 1.34695±0.00008 | 1.02647±0.00026 | 1.7643±0.0022 |

## S4. Effect of Crowding on Thermal Aggregation of Ph*b* in the Absence and in the Presence of HSPB5

The kinetic parameters of thermal aggregation of Ph*b* (0.4 mg/mL) both in the absence and presence of HSPB5 at 48 °C (0.03 M Hepes buffer, 0.1 M NaCl, 0.2 mM EDTA, pH 6.8) were obtained by the analysis of the data on dynamic light scattering of the samples (see the main text).

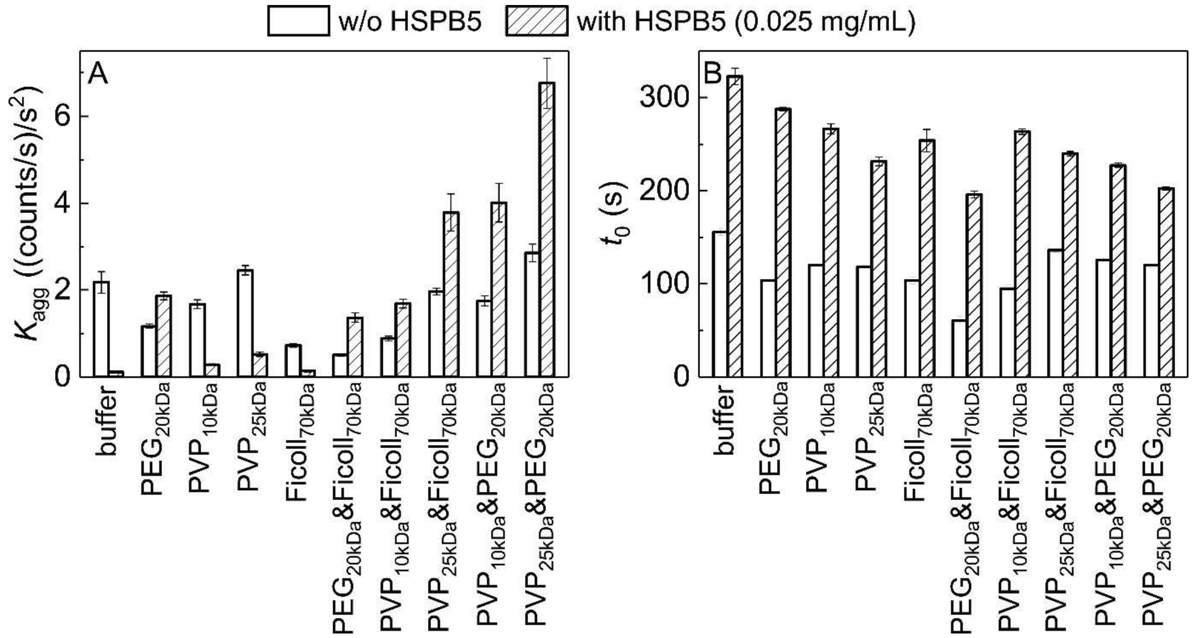


**Figure S3.** The effect of crowding on the kinetic parameters of the thermal aggregation of Ph*b* (0.4 mg/mL) at 48 °C (0.03 M Hepes buffer, 0.1 M NaCl, 0.2 mM EDTA, pH 6.8). Bar diagrams of $K_{agg}$ values (A) and the duration of the lag phase $t_0$ (B) in the absence (hollow bars) and presence (shaded bars) of HSPB5 (0.025 mg/mL). The concentration of Ficoll$_{70kDa}$ in the experiments is 75 mg/mL, the concentrations of the remaining crowders are 25 mg/mL.

## S5. The characteristics of HSPB5 preparation.

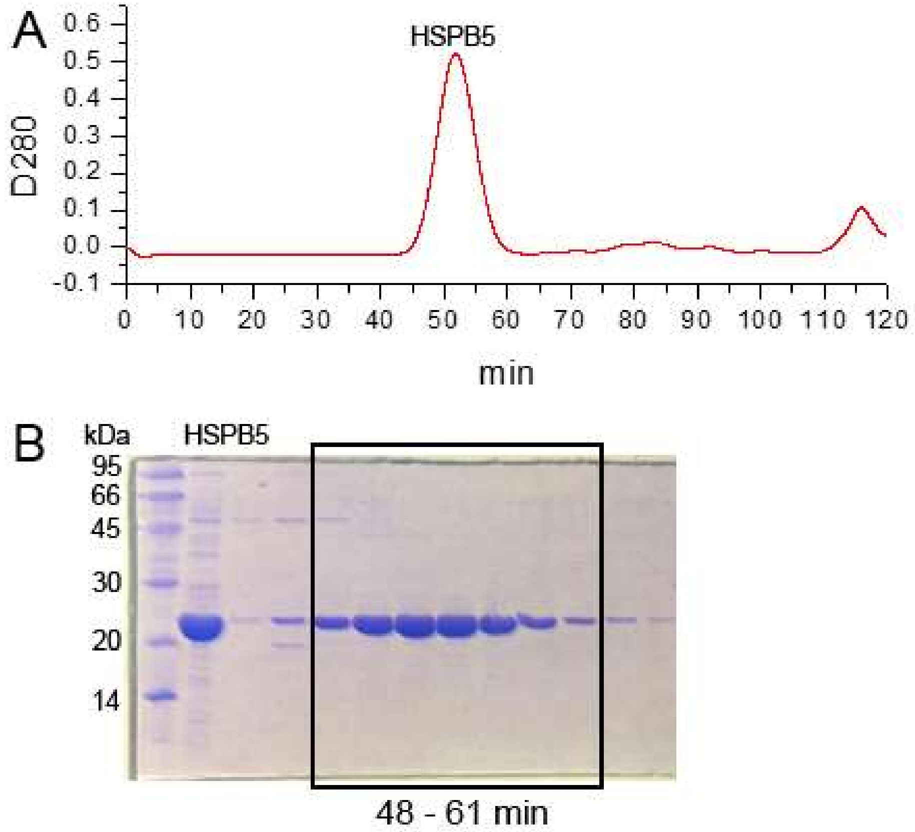


**Figure S4.** The characteristics of HSPB5 preparation. (A) The elution profile of HSPB5 obtained by SEC. (B) SDS-gel electrophoresis of the stock HSPB5 preparation (lane 2) and the fractions from the peak in panel A in the elution times interval 48–61 min (lanes 5–11). The MW standards are presented in the first lane with their mass in kDa indicated on the left.

© 2020 by the authors. Submitted for possible open access publication under the terms and conditions of the Creative Commons Attribution (CC BY) license (http://creativecommons.org/licenses/by/4.0/).
